# Integrin αvβ6 and transcriptional factor Ets-1 act as prognostic indicators in colorectal cancer

**DOI:** 10.1186/2045-3701-4-53

**Published:** 2014-09-02

**Authors:** Cheng Peng, Huijie Gao, Zhengchuan Niu, Ben Wang, Zhen Tan, Weibo Niu, Enyu Liu, Jiayong Wang, Jiuzheng Sun, Muhammad Shahbaz, Michael Agrez, Jun Niu

**Affiliations:** Department of Hepatobiliary Surgery, QiLu Hospital, Shandong University, Jinan, Shandong China; Health Science College, The State University of New York -Stony Brook University, Stony Brook, NY USA; Department of General Surgery, Jinan Central Hospital, Shandong University, Jinan, Shandong China; Newcastle Bowel Cancer Research Collaborative, Hunter Medical Research Institute, John Hunter Hospital and Faculty of Medicine and Health Sciences, The University of Newcastle, Callaghan, NSW Australia

## Abstract

**Background:**

Both transcriptional factor Ets-1 and integrin αvβ6 play an important role in the development and progression of cancer. The aim of our study was to investigate the expression of Integrin αvβ6 and Ets-1, two proteins’ correlation and their clinical significance in colorectal cancerous tissues.

**Results:**

The specimens were arranged into microarray using the immunohistochemistry method to investigate the expression of integrin αvβ6 and transcriptional factor Ets-1 in these tissues. Among the 158 tissue specimens, 36.07% were positive for αvβ6 expression, and 57.59% were positive for Ets-1 expression. There were obvious statistical differences existed regarding differentiation, N stage, M stage and TNM stage between αvβ6 and Ets-1 positively and negatively expressing tumors. The correlation analysis confirmed the expression of αvβ6 and Ets-1 were positively correlated in colorectal cancer. The Kaplan-Meier survival analysis showed that patients who were both αvβ6 and Ets-1 positive relapsed earlier than those who were both αvβ6 and Ets-1 negative; and the former group had much shorter survival time than the latter. And Cox model indicated that αvβ6 and Ets-1 were the independent prognostic factors (RR = 2.175, P = 0.012 and RR = 3.903, *P* < 0.001).

**Conclusions:**

The expression of αvβ6 and Ets-1 were positively correlated, and their expression degrees were associated with the differentiation, N stage, M stage and TNM stage of the tumors. Hence, the combination of αvβ6 and Ets-1 can be used as a prognostic marker in colorectal cancer, especially for the early stage.

## Introduction

Colorectal Cancer (CCR) is the most frequent gastrointestinal malignant tumor in the world. According to an estimate of International Agency for Research on Cancer (IARC), there will be about 1.2 million new colorectal patients all over the world every year, and the mortality rate will account for about 8% of all malignancies [1]. Generally, the CCR occurs more frequently in developed countries. The number of CCR incidence and mortality rates increased a lot in China in the past few years [2]. From 2006 to 2009, the CCR became the 3rd prevalent and 5th most malignant among the entire malignant tumor in China. Therefore, CCR seriously affected the human health [3].

αvβ6 is a special subtype of integrin that is expressed in epithelial cells only, and its major ligand is fibronectin (FN). In normal epithelial cells, the expression of αvβ6 is rare and can hardly be detected [4], but it increases substantially in response to injury and/or inflammation, or in epithelial tumors [5]. Our previous studies show that the *de novo* expression of the αvβ6 integrin has been shown to modulate several processes in colon carcinoma cells, including cell adhesion and spreading on fibronectin, proliferation within collagen gels, tumor growth, cell apoptosis and matrix metalloproteinase (MMPs) secretion [6, 7]. We also have suggested that the αvβ6 integrin is a prognostic indicator of gastric carcinoma, and αvβ6 would be a useful index to direct early therapy in order to prevent the spread of cancer [8].

Ets-1 is a kind of transcription factor which is present in species ranging from sponges to human. All family members contain an approximately 85 amino acid DNA binding domain, designated as the Ets domain. Ets-1 proteins bind to special purine-rich DNA sequences with a core motif of GGAA/T, and transcriptionally regulate in a number of viral and cellular genes. Thus, Ets proteins are an important family of transcription factors that control the expression of genes which are critical for several biological processes, including cellular proliferation, differentiation, development, transformation, and angiogenesis [9–11].

Although, many studies have been conducted about finding molecular markers as prognostic indicators for the malignant biological behavior of CCR; there is still a demand to find indicators or its combinations regarding the CCR development and progression. Since many papers have shown that both integrin αvβ6 and transcription factor Ets-1 participate in the regulation of malignant tumor biological behavior, therefore we performed the immunohistochemical assessment of integrin αvβ6 and transcription factor Ets-1. We expected these markers or their combinations would become the prognostic indicators in CCR.

## Results

### Follow up

All the specimens can be used to assess the expression of integrin αvβ6 and transcriptional factor Ets-1. 5-year follow-up was conducted among all the 158 patients, and the follow-up rate was 100%. Among these, 71 (44.9%) were confirmed cancer specific death within 5 years of prognosis and 87 (55.1%) were censored as their case follow up was discontinued or patients were alive beyond 60 months or died of reasons other than colon cancer.115 patients (72.8%) relapsed from cancer during 5-year follow-up, and 43 (27.2%) were censored. Follow-up based on patient’s history, physical examination, complete blood count, liver function tests, tumor markers monitoring, ultrasound scan of the abdomen every 3 months, abdominal pelvic CT scans and colonoscopy every 6 months. Patients with high-risk factors were encouraged to take adjuvant chemotherapy. Patients with liver metastasis tumor accepted the radiofrequency ablation (RFA) or super γ knife radiotherapy. 87 patients received fluorouracil-based adjuvant chemotherapy, and 28 patients received the RFA or radiotherapy.

### Expression of αvβ6 and Ets-1 in the tissue specimens and clinical character

**Figure 1:**
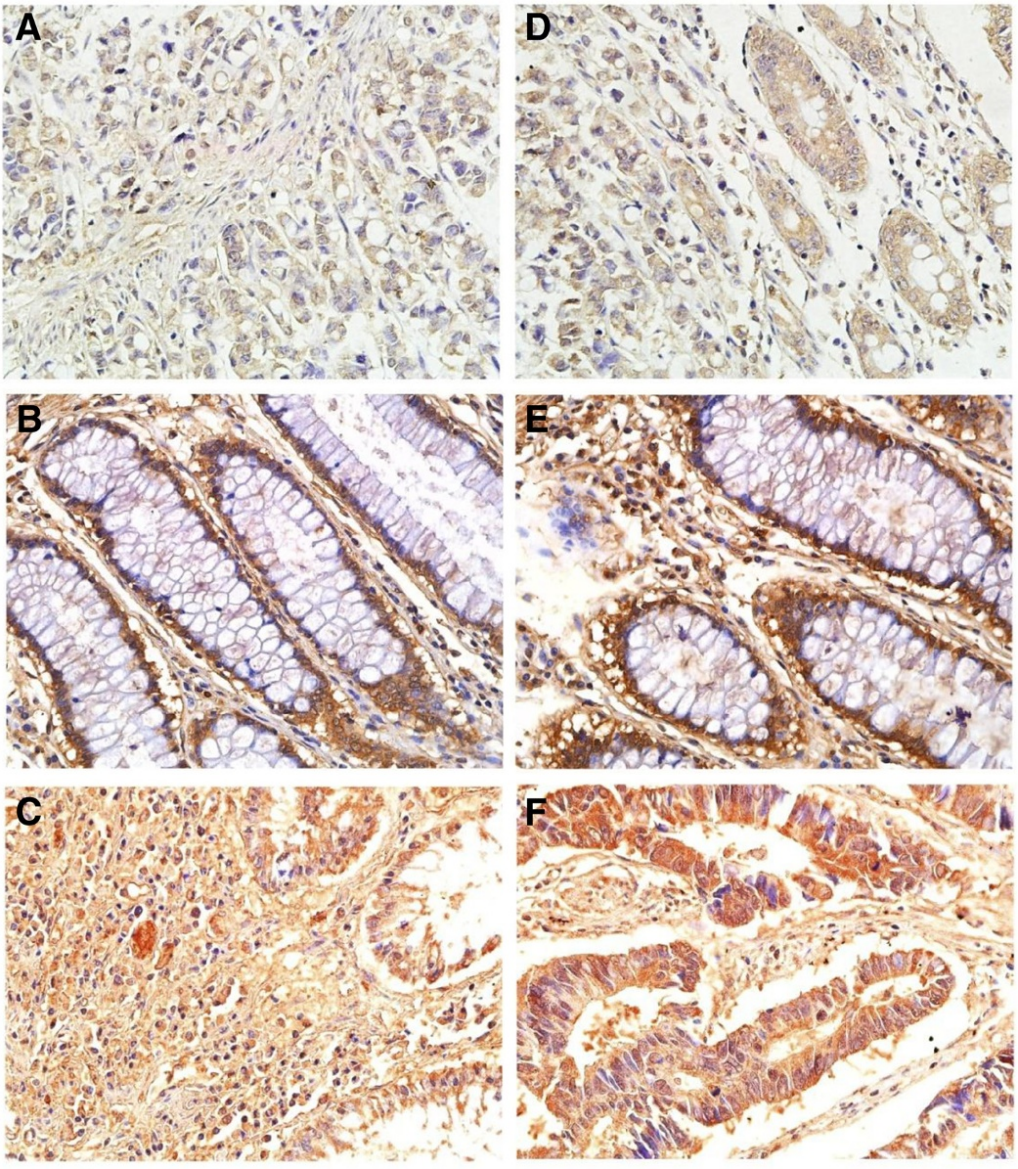
**Images of αvβ6 and Ets-1 immunohistochemistry staining in human colorectal cancer samples.** αvβ6 staining was observed predominantly in the cell membrane. **A-C** the immunohistochemistry photomicrographs of integrin αvβ6. **A**: integrin αvβ6 weak staining, **B**: integrin αvβ6 moderate stainning, **C**: integrin αvβ6 high staining; Ets-1 mainly exhibited a nuclear and cytoplasmic immunostaining in tumor cells. **D-F** the immunohistochemistry photomicrographs of transcriptional factor Ets-1. **D**: Ets-1 weak staining, **E**: Ets-1 moderate staining, **F**: Ets-1 high staining.

In the 158 tissue specimens, 57 (36.07%) were positive for αvβ6 expression, and 91 (57.59%) were positive for Ets-1 expression. Clinicopathological features and αvβ6 & Ets-1 expression of all specimens were expounded in details in Table 1. And the expression levels of αvβ6 and Ets-1 in 10 samples of normal colon mucosa were both negative. A following analysis showed that there was no evidence of a statistical difference between αvβ6 or Ets-1 positive and negative tumors regarding age, gender, location, tumor size, Duke’s phase, T stage, and histopathological type, but significant statistical differences existed regarding differentiation, N stage, M stage and TNM stage. αvβ6 and Ets-1staining patterns in the tissue specimens are shown in Figure 1. High αvβ6 expression was detected at the invading edge of the tumor, and staining was observed predominantly in the cell membrane. Ets-1 mainly exhibited a nuclear and cytoplasmic immunostaining in tumor cells.

**Table 1:** **Patients characteristic and the expression of intgerin αvβ6 and transcriptional factor Ets-1**

Factor	N	αvβ6			***P***	Ets-1			***P***
		Negative	Low	High		Negative	Low	High	
**Age**									
<60 Y	62	34	16	12		27	23	12	
>60 Y	96	67	14	15	0.127	40	36	20	0.964
**Gender**									
Male	115	75	20	20		49	43	23	
Female	43	26	10	7	0.705	18	16	9	0.991
**Location**									
Colon	127	83	24	20		55	48	24	
Rectum	31	18	6	7	0.641	12	11	8	0.688
**Tumor size**									
<2 cm	15	9	4	2		6	6	3	
2 ~ 5 cm	109	70	22	17		47	40	22	
>5 cm	34	22	4	8	0.624	14	13	7	0.999
**Differentiation**									
Well	28	22	4	2		15	9	4	
Moderate	93	67	16	10		41	37	15	
Poor	27	11	8	8		10	10	7	
Unknown	10	1	2	7	0.000	1	3	6	0.035
**Duke’s phase**									
A + B	92	59	20	13		40	34	18	
C + D	66	42	10	14	0.366	27	25	14	0.942
**T stage**									
T1	48	31	8	9		19	18	11	
T2	70	42	16	12		28	30	12	
T3	33	24	4	5		18	9	6	
T4	7	4	2	1	0.851	2	2	3	0.470
**N stage**									
N0	68	56	6	6		39	21	8	
N1	67	33	18	16		22	28	17	
N2	23	12	6	5	0.001	6	10	7	0.015
**M stage**									
M0	130	93	24	13		60	52	18	
M1	28	8	6	14	0.000	7	7	14	0.000
**TNM stage**									
I&II	66	56	5	5		32	23	11	
III	64	37	19	8		28	29	7	
IV	28	8	6	14	0.000	7	7	14	0.000
**Histopathological type**									
Tubular adenocarcinoma	134	85	26	23		58	52	24	
Mucinous adenocarcinoma	15	10	2	3		6	4	5	
Other type	9	6	2	1	0.955	3	3	3	0.826

### Correlation between the αvβ6 and Ets-1expression

In the 158 tissue specimens, 101 (63.92%) were negative for αvβ6 expression, 30 (18.99%) were low positive for αvβ6 expression, 27 (17.09%) were high positive for αvβ6 expression; 67 (42.41%) were negative for Ets-1 expression, 30 (37.34%) were low positive for Ets-1 expression, 32 (20.25%) were high positive for Ets-1 expression. The expression of αvβ6 and Ets-1 were positively correlated (*r* = 0.711, *P* = 0.000), Specific data was shown in Table 2.

**Table 2:** **Expression correlation of intgerin αvβ6 and transcriptional factor Ets-1**

αvβ6 expression	Ets-1 expression		
	Negative	Low	High
Negative	64	35	2
Low	2	19	9
High	1	5	21

### Expression of both αvβ6 and Ets-1 predicts relapse and patients survival

**Figure 2:**
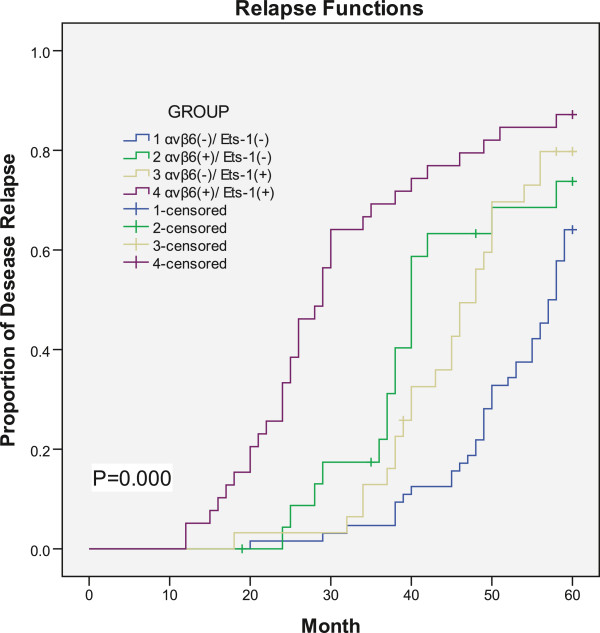
**Disease relapse time is also shown by Kaplane-Meier survival analysis using a Log-rank test.** The samples were grouped according to αvβ6 and Ets-1 expression. (*P* = 0.000).

**Figure 3:**
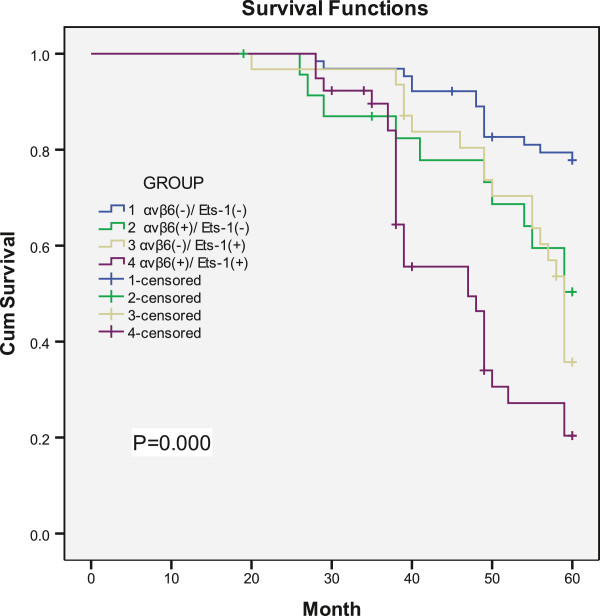
**Patients survival time is shown by Kaplane-Meier survival analysis using a Log-rank test.** The samples were grouped according to αvβ6 and Ets-1 expression. (*P* = 0.000).

According to the expression of αvβ6 and Ets-1, all patients were divided into four categories: both αvβ6 and Ets-1 positive group (Group 1), αvβ6 positive with Ets-1 negative group (Group 2), αvβ6 negative with Ets-1 positive group (Group 3), and both αvβ6 and Ets-1 negative group (Group 4), respectively. The Kaplan-Meier survival analysis showed that patients who were both αvβ6 and Ets-1 positive relapse earlier (32.59 ± 2.37) than those who were in Group 2 (43.82 ± 2.57), Group 3 (46.68 ± 1.83), and Group 4 (53.30 ± 1.10) (*P* = 0.000).And patients who were in either αvβ6 or Ets-1 positive (Group 2 and 3) also relapsed earlier than those who were both αvβ6 and Ets-1 negative (*P* < 0.0001) (Figure 2). Likewise, the Kaplan-Meier survival analysis showed that patients who were both αvβ6 and Ets-1 negative live longer (56.82 ± 0.95) than those who were both αvβ6 and Ets-1 positive (46.23 ± 1.71), and those who were in Group 2 (52.31 ± 2.41) or Group 3 (53.62 ± 1.71) (*P* < 0.0001). And patients who were in Group 2 or Group 3 live shorter than those who were both αvβ6 and Ets-1 negative (*P* < 0.0001) (Figure 3).

### Univariate and multivariate analysis for prognosis of patients with colon cancer

The Cox proportional hazards regression model were used to conduct Univariate and multivariate data analyses to determine the prognostic value of αvβ6 and Ets-1 expression. Positive expression of αvβ6 and Ets-1, as well as senior grade of N stage, M stage, and TNM stage were the factors to predict a poor prognosis in univariate analysis (Shown in Table 3). Then the variables with P < 0.05 were chosen to conduct multivariate analysis, we revealed that positive expression of αvβ6 and Ets-1 were unfavorable independent prognostic factors (RR = 2.175, *P* = 0.012 and RR = 3.903, *P* < 0.001, respectively) (Shown in Table 3).

**Table 3:** **Univariate and multivariate analysis of association of clinicopathologic features with survival time**

Variable	Univariate analysis			Multivariate analysis		
	Relative risk	95% CI	P value	Relative risk	95% CI	P value
**Age at diagnosis**	0.996	0.980,1.011	0.583			
**Gender**						
Male	1.000(Ref.)	-	-			
Female	1.053	0.651,1,702	0.834			
**Location**						
Colon	1.000(Ref.)	-	-			
Rectum	1.014	0.628,1.638	0.953			
**Tumor size**			0.064			
<2 cm	1.000(Ref.)	-	-			
2 ~ 5 cm	0.592	0.282,1.245	0.167			
>5 cm	1.356	0.798,2.302	0.260			
**Duke’s phase**						
A + B	1.000(Ref.)	-	-			
C + D	1.460	0.877,2.432	0.146			
**T stage**			0.386			
T1	1.000(Ref.)	-	-			
T2	1938.169	0.000,>10 5	0.912			
T3	2612.435	0.000,>10 5	0.909			
T4	3765.785	0.000,>10 5	0.905			
**N stage**			0.010			0.721
N0	1.000(Ref.)	-	-	1.000(Ref.)	-	-
N1	2.202	1.302,3.727	0.003	0.697	0.261,1.859	0.470
N2	1.845	0.921,3.696	0.084	0.649	0.221,1.903	0.431
**M stage**						
M0	1.000(Ref.)	-	-	1.000(Ref.)	-	-
M1	3.430	2.019,5.828	<0.001	1.998	0.432,9.235	0.376
**TNM stage**			<0.001			0.709
I	1.000(Ref.)	-	-	1.000(Ref.)	-	-
II	1.421	0.427,4.734	0.567	1.599	0.479,5.341	0.446
III	2.697	0.804,9.049	0.108	1.846	0.384,8.887	0.444
IV	6.005	1.777,20.296	0.004	#	#	#
**Differentiation**			0.257			
Well	1.000(Ref.)	-	-	1.000(Ref.)	-	-
Moderate	1.606	0.802,3.216	0.181			
Poor/unknown	1.794	0.889,3.620	0.103			
**Ets-1**						
Negative	1.000(Ref.)	-	-	1.000(Ref.)	-	-
Positive	4.913	2.792,8.645	<0.001	3.903	1.945,7.832	<0.001
**αvβ6**						
Negative	1.000(Ref.)	-	-	1.000(Ref.)	-	-
Positive	3.775	2.324,6.133	<0.001	2.175	1.190,3.974	0.012

#: TNM stage IV is linearly dependent on M stage M1.

## Discussion

Colorectal cancer has a higher morbidity and mortality in the world. Despite improvements made in screening and treatments during the past years, the clinical outcome of colon cancer remains unsatisfactory. Understanding the molecular mechanism of tumorigenesis would contribute to treatment of patients with this disease [12].

The data in the present study indicated that the total expression percentage of integrin αvβ6 and Ets-1 in colorectal cancer was 36.07% and 57.59% respectively. The expression level of αvβ6 and Ets-1 was associated with the differentiation, N stage, M stage and TNM stage of the tumors. Correlation analysis showed that the expression of αvβ6 and Ets-1 were positively correlated, and their strong stains were always detected at the invading edge of the tumor, weak or no stain always in the center of the tumor. These findings about integrin αvβ6 expression were similar with our previous study in gastric carcinoma [8], and Ets-1 expression pattern and its role look like Ito [13]. The phenomenon above could be explained as following: High cell density in tumor induces integrin αvβ6 and Ets-1 expression, which promote MMP-9 secretion for colon cancer cells,increasing the degradation of extracellular matrix (ECM),which constitutes the molecular biological basis for a self-perpetuating system of tumor invasive growth in colon cancer progression.

In the present study, survival analysis showed that patients who were both αvβ6 and Ets-1 positive relapsed earlier than others, and their survival time was also obviously shorter. Based on these data, we confirmed that the concomitant expression of αvβ6 and Ets-1 in colorectal cancer cells could be used as an independent predictor to determine early relapse, metastases and prognosis in CRC patients. The important conclusion of this study is similar with our previous study [14] and Zsuzsanna [15].

We have previously shown a direct linkage between ERK2 and the cytoplasmic domain of β6, and have delineated the binding domain for ERK2 within the cytoplasmic tail of the β6-integrin subunit, ^746^EAE*RSKAKWQTGTNPLYR*G^764^ (the ERK2 binding sequence is italic) [16]. Through this physical interaction, integrin αvβ6 transmitted a growth-enhancing signal to cancer cells. *In vivo* studies of colon cancer xenografts expressing a β6-mutant lacking the ERK2 binding domain indicated that deletion of the β6-ERK2 binding motif greatly compromised tumor growth. Taken all the factors into consideration, these results suggested that tumor growth was, at least in part, dependent on direct αvβ6-ERK binding. Meanwhile, we also have known that in colorectal cancer cells αvβ6-ERK2 direct linkage could increase the phosphorylation level of ERK2, and ERK2 conformation would be changed as soon as binding with αvβ6. To some extent, this process helped ERK2 to be phosphorylated easily, and protected phosphorylated ERK2 not to be dephosphorylated [17]. The effective targets of phosphorylated-ERK2 were localized in the nucleus. Unfortunately, the nuclear factors which could be regulated by phosphorylated-ERK2 still remained unclear.

Park showed that the expression of Ets-1 has intimate correlation with tumor cell invasion and metastasis. Ets-1 and MMPs co-expressed in colon cancer and other malignant tumor, and its expression degree would be increased with tumor invasion and metastasis extent accordingly, but its expression was very low in benign and non-invasive tumor [18]. Hashiya confirmed that Ets-1 played an important role in promoting the tumor angiogenesis through regulating angiogenesis factor, such as VEGF, Ang2, and so on [11]. Other studies showed that Ets-1 could induce the tumor cell apoptosis through regulating cell cycle via changing the expression of p21, p53, cyclinD1, c-Fos in tumor cells [19–21].

In this study, we confirmed that the αvβ6 and Ets-1expressions were positively correlated, and they always expressed in invading edge of the tumor, and their expression degree were associated with the differentiation, N stage, M stage and TNM stage of the tumors. So we thought that Ets-1 was likely to be the nuclear factor of ERK2. Perhaps, we proposed that integrin αvβ6 regulated the cancer cell malignant biological behavior through αvβ6—ERK—Ets-1 signal pathway.

Importantly, Ets-1 could transcriptionally regulate the expression of integrin beta6, which has been reported by Bates et al. [22]. Thus, a signal loop involving Ets-1 and αvβ6 probably exists to play a key role in tumor cellular growth and migration. Of course, our hypotheses need demonstrating in our future work.

We have already demonstrated that β6-integrin is associated with chemo resistance in colon cancer, and β6-integrin could induce 5-FU resistance through the ERK/MAP kinase pathway and the β6-ERK2 direct binding [23]. In our another study, we have also proved that Norcantharidin (NCTD), as an anti-cancer traditional chinese medicine, could decrease αvβ6 expression and inhibit ERK phosphorylation in HT-29 cells [24]. In the present study, we found combination of αvβ6 and Ets-1 could favor colon cancer prognosis. These results indicate β6-integrin might be a novel therapeutic target in colon cancer therapy, and it reminded us that if positive αvβ6 and Ets-1 were found in patients with colon cancer of early stage, compared with negative, we would take more aggressive chemotherapy, or more frequent reexamination. However, patients in advanced stage have a worse prognosis, whatever aggressive therapy to take. Therefore, we should take earlier diagnosis and treatment of cancer, and different therapy methods could be applied in patients with distinct gene expression patterns.

To Date, based on this study results and other previous conclusions, we think that co-expression of αvβ6 and Ets-1 could serve as a significant prognositic indicator for colorectal cancer, and their interaction might play an important role in cancer progression. Therefore, any key molecule in αvβ6—ERK—Ets-1 signal pathway, or even the potential signal loop, can be used as an attractive therapeutic candidate for CRC. This new study is encouraging. However, further research is needed to confirm the findings and to establish the relationship between the αvβ6 and Ets-1 as a significant prognositic indicator for colorectal cancer.

## Materials and methods

### Antibodies and reagents

The mouse anti-human monoclonal antibody 6.2A1 (IgG1) against integrin αvβ6 was obtained from Biogen (Cambridge, USA), monoclonal antibody A0478 (IgG1) against Ets-1 was obtained from Bio-tech (Sunnyvale, California, USA), and biotinylated rabbit anti-mouse IgG was obtained from Dako (Copenhagen, Denmark).

### Human tissue specimens

158 cases of colorectal carcinoma specimens and endoscopic colon biopsy samples of normal colon mucosa (n = 10) which obtained from January 2006 to June 2008, were selected randomly from Qilu Hospital of Shandong University (Jinan, Shandong Province, China). Those colorectal carcinoma and normal colorectal mucosa specimens were formalin-fixed and paraffin-embedded. The informed consent was obtained from all the patients or their relatives. The study complied with the requirements of The Ethics Committee of Qilu Hospital, Shandong University.

### Immunohistochemistry

Immunohistochemistry was performed on 5 micrometer sections of the FFPE routine sections and tissue microarray (TMA) analysis using monoclonal mouse antibodies against αvβ6 (6.2A1) and Ets-1 (A0478) was applied respectively. The sections were deparaffinized and hydrated, and the heat induced epitope retrieval was performed using Borg decloaking high pH buffer in the Biocare decloaking chamber. Endogenous peroxidase’s activity was blocked with 3% hydrogen peroxide. The slides were first incubated with an avidin-biotin kit, followed by incubations with the αvβ6 primary antibody (1:500 dilutions) overnight at 4°C, streptavidin-horseradish peroxidase, and Betazoid Diaminobenzidine for color development. Negative controls were prepared by using the identical concentration of mouse immunoglobulin IgG1 (Dako). The next day, biotinylated anti-mouse IgG (1:200) was applied to the slides, which were subsequently treated using horseradish peroxidase (HRP)-labelled streptoantibiotin (Dako) for 15 min. The slides were counterstained with Dako Hematoxylin, rinsed with water, and then dehydrated with alcohol and xylene, and coverslipped. Appropriate controls were included. All incubations were done at the room temperature.

### Assessment of αvβ6 and Ets-1 scoring in the tissue sections

αvβ6 stain was mainly on the internal surface of the tumor cell membrane. αvβ6 and Ets-1 stain were evaluated independently by two pathologists who were blinded to patients’ prognosis. Disagreements were resolved by discussion in a meeting which was held to obtain the final results. The proportion score represented the estimated fraction of positive staining cells (0, 0%; 1, <20%; 2, 20 ~ 50%; 3, 51 ~ 75%; 4, > 75%). Intensity of the staining: 0, no staining; 1, weak staining, pale brown; 2, moderate staining, brown; 3, strong staining, dark brown. We took into account both the intensity and the proportion of positive cells to give a semi quantitative estimate of the expression levels of antigen in the tissue core. Added the two scores, the last score of every section was obtained. We defined the staining class according to the last score as follows: tumors with a final score < 2 were designated as negative expression, 2 ~ 4 as low (weak) expression, and ≥5 as high (strong) expression. Both low and high expressions were graded as αvβ6 and Ets-1 positive.

### Patients and follow-up

From January 2006 to June 2008, 158 patients who underwent curative resection by the same surgical team for pathologically confirmed colon cancer at the Department of Pathology of Qilu Hospital (Shandong University, China) were divided into three groups (both αvβ6 and Ets-1 positive group, both αvβ6 and Ets-1 negative group, and either αvβ6 or Ets-1 positive group) to investigate disease relapse and survival time. In our protocol for the follow-ups, all patients were followed every 3 months in the first year and at least every 6 months afterwards. They were examined with regular monitoring of metastasis by abdomen ultrasonography or computed tomography (CT) studies of the peritoneal organs and lymph nodes. New relapse lesions were diagnosed based on the typical image findings from CT scans.

### Statistical methods

Associations between immunohistochemical scores and clinicopathologic variables of tissue specimens were evaluated by *χ*^2^ test. Expression correlation of αvβ6 and Ets-1 was assessed using bivariate correlation analysis by *χ*^2^ test. Disease relapse time was referred to as the time from the date of surgery to colorectal cancer-related disease appearance, including local recurrence, liver metastasis, peritoneal metastasis, significantly elevated tumor markers, and etc. Survival time was measured as the time from the date of surgery to disease-related death, and those who died from other reasons or were still alive when they were last seen were censored. Survival analysis was carried out using the Kaplane-Meier survival and Cox model analysis. *P* < 0.05 was considered statistically significant. Graphs showed the mean ± SD of data from a representative experiment; data were representative of at least three experiments with comparable results.
